# Effects of storage time and temperature on highly multiparametric flow analysis of peripheral blood samples; implications for clinical trial samples

**DOI:** 10.1042/BSR20203827

**Published:** 2021-02-26

**Authors:** Amelia Jerram, Thomas V. Guy, Lucinda Beutler, Bavani Gunasegaran, Ronald Sluyter, Barbara Fazekas de St Groth, Helen M. McGuire

**Affiliations:** 1 Discipline of Pathology, Faculty of Medicine and Health, The University of Sydney, Camperdown, NSW 2050, Australia; 2 Immunology and Cell Signalling Group, Illawarra Health and Medical Research Institute, Wollongong, NSW 2522, Australia; 3 Molecular Horizons and School of Chemistry and Molecular Bioscience, University of Wollongong, Wollongong, NSW 2522, Australia; 4 Ramaciotti Faculty for Human Systems Biology, The University of Sydney, Camperdown, NSW 2050, Australia

**Keywords:** Blood processing, cytometry, immunophenotyping, neutrophil

## Abstract

We sought to determine the effect of time and temperature of blood sample storage before preparation of human peripheral blood mononuclear cells (PBMCs) by Ficoll-hypaque density gradient centrifugation. Blood samples from healthy donors were stored at room temperature (RT) or refrigerated at 4°C before preparation of PBMCs. Cell yield and viability, and proportions of major cell populations within PBMCs, as determined by fluorescence flow cytometry, were assessed for both fresh and cryopreserved samples. Highly multiparametric mass cytometry was performed on cryopreserved PBMCs. We found that refrigeration had marked negative effects on subsequent PBMC yield. Storage at RT led to co-purification of low density neutrophils with PBMCs, but had no detectable effects on the proportions of multiple cell subsets including, but not limited to, monocytes, NK cells, B cells, Treg cells, and naïve, central memory and effector memory CD4^+^ and CD8^+^ T cells and CD45RA-positive terminal effector CD8^+^ T cells. Expression of a number of cell surface receptors, including CXCR5, CCR6, CXCR3 and TIGIT, but not CD247 was reduced after RT storage before PBMC preparation, and this effect correlated with the degree of low density neutrophil contamination. As such, when PBMC preparation cannot be undertaken immediately after blood draw, storage at RT is far superior to refrigeration. RT storage leads to neutrophil activation, but does not compromise measurement of PBMC subset distribution. However caution must be applied to interpretation of cytometric measurements of surface molecules such as chemokine receptors.

## Introduction

New immune-directed measures have revolutionised the way cancer is treated [1], but many patients fail to mount clinically beneficial responses. Blood immunophenotyping analysis using flow cytometry is an important element in understanding patient response to immunotherapy [2,3]. Such analyses are often performed on peripheral blood mononuclear cells (PBMCs), which consist of lymphocytes and monocytes separated from other blood components, such as granulocytes, platelets and red blood cells, using density gradients [4]. To ensure the validity of immune profiles derived from immunophenotypic analyses, blood samples would ideally undergo density gradient separation immediately post-venesection. However, this is not always feasible due to the location of clinical trial centres relative to central research laboratories. It is therefore critical to understand how modification of best practice in blood processing affects immunophenotyping data.

Both time-to-processing and temperature-before-processing may introduce artefacts. The effects of different whole blood storage conditions on PBMCs have been analysed for a limited number of immune populations and cellular markers [5–12]. These studies have not yet been extended to include highly multiparametric immunophenotyping techniques such as mass cytometry.

Refrigeration of blood does not generally introduce variation in full blood counts [13,14], but has been reported to modify representation of multiple lymphocyte subsets within PBMCs [5,15,16]. The effect of low ambient temperatures, such as those encountered during shipping in cold climates, has received less attention, but is highly relevant for design of multicentre trials. In a large study in which a temperature gauge was included with each blood sample, reduced PBMC recovery after density separation was shown to correlate with exposure to lower temperatures during shipping [9]. This study also demonstrated that exposure to 15°C for as little as 2 h compromised PBMC recovery and viability. The effect was more apparent for CD8 T cells compared with CD4 T cells, leading to an increased CD4/CD8 ratio after 15°C storage.

When blood samples are held at room temperature (RT), generally considered to be in the range of 20–22°C, time-to-processing is another factor that may impact PBMC quality. There is as yet no clear consensus regarding the effect of RT storage on cell recovery, viability and lymphocyte subset distribution [11,12]. Extended RT storage has been reported to lead to an increase in low density neutrophils that co-purify with PBMCs [7,8] due to an activation-induced reduction in neutrophil density. A similar density shift has been described in the blood of patients suffering from autoimmune and inflammatory diseases [17,18] and late stage cancer [19], although a proportion of these low density neutrophils may be immature band forms (due to a so-called ‘left shift’ in haemopoiesis) rather than activated mature neutrophils [17,18,20].

The present study aimed to provide a comprehensive analysis of immune populations in PBMCs when blood samples were exposed to different conditions prior to Ficoll-hypaque density gradient separation. An initial pilot study was performed to determine the effects of storage time at 4°C on lymphocyte and monocyte composition of cryopreserved PBMCs. This led us to perform a more comprehensive study assessing the effects of storage at 4°C versus RT for 0, 6 or 24 h. We performed differential blood counts, cytocentrifugation for visualisation of cell morphology, and fluorescence and mass-based cytometry. For samples stored at RT prior to processing, fluorescence flow cytometry was used to compare the distribution of major cell populations in unmanipulated blood and freshly isolated PBMCs. PBMCs were also cryopreserved, then thawed and analysed using mass flow cytometry with a broad immunophenotyping panel, to identify more subtle changes in proportions of cell subsets and expression of a comprehensive range of immune markers.

## Methods

### Study participants

For the pilot study, healthy volunteers (*n*=5) were recruited with informed, written consent. The protocol was conducted under University of Wollongong ethics protocol HE12/219. For the main study, eight healthy volunteers, four males and four females, aged 21–70 years, were recruited. All participants gave written informed consent, and the study was approved by the Sydney Local Health District Ethics Committee.

### Blood collection and storage before processing

For the pilot study, 20 ml of blood was taken at a single blood draw and immediately transferred into four individual 5-ml EDTA vacutainer venous blood collection tubes (BD Biosciences, San Jose, CA). At 0, 6, 12 and 24 h post-venesection, one tube per donor underwent density gradient separation and cryopreservation of PBMCs. The 6-, 12- and 24-h time point samples were kept refrigerated at 4°C between venesection and density gradient separation. For the remainder of the study, a total of 35 ml of blood was collected at a single blood draw from each of the participants via venepuncture and immediately transferred into five individual BD Vacutainer tubes containing sodium heparin (BD Biosciences, San Jose, CA). One tube per participant was processed within 20 min. The remaining four tubes were stored vertically without shaking at either 4°C or RT (approx. 20°C), for 6 or 24 h before processing. Five of the eight study participants contributed a second blood donation of five tubes which were treated in an identical manner. The sample from one of the original eight donors was of insufficient volume for five tubes and the 4°C storage arm was omitted.

### Ficoll-Paque gradient separation and cryopreservation

At the time of blood processing, samples were transferred to a 50 ml tube, mixed with an equal volume of phosphate buffered saline (PBS) and then underlaid with 1.077 g/ml Ficoll Paque™ PLUS (GE Healthcare Bio-sciences, Uppsala, Sweden) before centrifugation at 500×***g*** for 30 min with the brake off. PBMCs were isolated from the interface between the Ficoll and plasma layers, transferred to a new tube and washed with 3× volume of PBS supplemented with 5% foetal bovine serum (FBS) (SAFC, Missouri, U.S.A.). In cases where a clear monolayer of cells was not observed at the Ficoll interface, 5 ml of liquid was harvested from the interface and processed. Washed PBMCs were cryopreserved at −80°C in freezing medium containing 10% dimethyl sulphoxide (DMSO), 80% RPMI (Lonza, Basel, Switzerland) and 10% FBS (SAFC, U.S.A.) in a Mr. Frosty™ freezing container (Thermo Fisher Scientific, Massachusetts, U.S.A.) allowing a rate of cooling of approximately −1°C/min to be achieved.

### Cell counts using Sysmex XP-300™ Automated Hematology Analyzer

Sixty microlitres aliquots of whole blood and post-Ficoll PBMCs in RPMI were analysed using the Sysmex XP-300™ Automated Hematology Analyzer (Sysmex, Kobe, Japan). Analysis was performed in triplicate for each sample and the average of the three counts was taken. The analyser outputs white blood cell (WBC) count/ml, plus neutrophil, lymphocyte and monocyte counts/ml when clearly distinct cell populations are present. When the resistive pulse sensing properties of cells have changed (for example, after storage and PBMC preparation), the analyser may not be able to resolve neutrophils, lymphocytes and monocytes and will generate only a white cell count.

### Fluorescence flow cytometry

For the pilot study, cryopreserved PBMC samples were thawed, up to 1 × 10^6^ cells were stained with 50 µl of an antibody cocktail comprising anti-CD3 (clone UCHT1, BD biosciences), -CD4 (RPA-T4, BD biosciences) and -CD14 (M5E2, BD biosciences) plus Zombie-Near IR live/dead (Biolegend) and data were collected on a BD Biosciences LSRFortessa™ X-20.

For analysis of whole blood samples stored at RT, 1 μl of undiluted Zombie NIR stain was added to 100 μl whole blood and incubated in the dark for 10 min. Fifty microlitres of antibody cocktail comprising anti-CD3 (clone UCHT1, BD biosciences), -CD4 (RPA-T4, BD biosciences), -CD8 (HIT8a, BD biosciences), -CD19 (HIB19, BD biosciences), -CD56 (NCAM16.2, BD biosciences), -CD14 (M5E2, Biolegend) and -CD16 (3G8, BD biosciences) was added and incubated at RT for 30 min. Two millilitres of FIX/LYSE solution (Invitrogen) was added for 20 min at RT, then samples were spun at 500×***g*** for 5 min before washing in FACs buffer (PBS, 5% FCS and 0.05% sodium azide) and resuspended in 500 µl FACs buffer for acquisition on a 5-laser BD Biosciences LSR-II.

For analysis of freshly isolated PBMCs, 500000 cells were washed twice with PBS to remove any protein present in the medium before addition of 100 μl PBS containing a 1:750 dilution of Zombie NIR stain. The mixture was incubated in the dark for 10 min, then cells were washed with PBS and once with FACs buffer before adding 50 µl of an antibody cocktail comprising anti-CD3, -CD4, -CD8, -CD19, -CD56, -CD14 and -CD16 (clones and suppliers as per whole blood analysis above) and incubation at RT for 30 min. Cells were fixed for 10 min in 1% paraformaldehyde (PFA), washed and resuspended in 500 µl FACs buffer before data acquisition on a 5-laser BD Biosciences LSR-II.

All fluorescence flow data were analysed using FlowJo (version 10.6.1) after compensation calculated from single stained bead controls acquired immediately before the cell samples.

### Cytocentrifugation

For a visualisation of live PBMCs isolated for flow cytometry, aliquots of freshly isolated PBMCs were centrifuged on to slides using a Tharmac Cellspin I at 800RPM for 5 min. The slides were air dried, fixed for 20 s fixation with 100% methanol and stained with Differential Quik Stain Kit (Modified Giemsa) (Electron Microscopy Sciences, Pennsylvania, U.S.A.) for 2 × 20 s. Slides were subsequently washed with water and imaged on an Olympus BX-51 microscope (Olympus, Tokyo, Japan) using a DP-70 camera and DP manager 2.2.1 software (Olympus).

### Mass cytometry

Cryopreserved PBMCs were quickly thawed in a 37°C water-bath and washed with 10 ml RPMI-1640 (Lonza) containing 10% FCS, and 1:10000 Pierce™ Universal cell nuclease (Thermo Fisher Scientific, U.S.A.). Trypan Blue (Gibco, Massachusetts, U.S.A.) staining was then performed to determine the number of live cells using a haemocytometer. Cells were counted twice by two different operators and the average count was calculated. A total of 600000 cells were stained with metal-tagged antibodies. In cases where cell count was lower than 600000, all cells were stained.

All antibodies were validated, pre-titered and supplied in per-test amounts by the Ramaciotti Facility for Human Systems Biology Mass Cytometry Reagent Bank. Reagent bank antibodies were purchased as unlabelled antibodies in a carrier-protein-free (from various suppliers as listed below for individual markers) and conjugated by the Ramaciotti Facility for Human Systems Biology with the indicated metal isotope using the MaxPAR conjugation kit (Fluidigm, South San Francisco, CA) according to the manufacturer’s protocol.

Cells were barcoded according to time delay in PBMC processing by incubation with anti-CD45 antibodies (clone HI30, Biolegend) conjugated with Palladium isotopes ^104^Pd (0 h), ^106^Pd (6 h) or ^108^Pd (24 h), for 30 min at 4°C before washing twice in CyFACs buffer (PBS, 0.02% sodium azide, 0.5% BSA and 2 mM EDTA). The three samples from each donor were then combined into a single tube so that signal intensity for each donor could be accurately quantitated as a function of time. The next step was to expose the cells to cisplatin (Fluidigm, U.S.A.) which allows for discrimination of live and dead cells. After the cisplatin stain, cells were washed and stained with 30 µl surface antibody cocktail, pre-filtered through a 0.1-µm centrifugal filter unit (Merck Millipore, Massachusetts, U.S.A.) for 30 min on ice. The surface staining cocktail detected the major cell populations: T cells (stained for expression of CD3 (clone UCHT1, Biolegend), CD4 (RPA-T4, Biolegend), CD8 (RPA-T8, Biolegend), CD45RA (HI100, Biolegend), CD45RO (UCHL1, Biolegend), CD25 (M-A251, Biolegend), CD127 (A019D5, Biolegend)), B cells (stained for CD19 (HIB19, Biolegend) and CD20 (2H7, Biolegend)), NK cells (stained for CD56 (REA196, Miltenyi Biotec)), monocytes (stained for HLA-DR (L243, Biolegend), CD14 (M5E2, BD biosciences) and CD16 (3G8, BD biosciences)) and neutrophils (stained for CD66b (G10F5, BD biosciences)). The cocktail contained additional antibodies recognising differentiation markers (IgD (IA6-2, BD biosciences), CD27 (M-T271, BD biosciences)) chemokine receptors (CCR6 (11A9, BD biosciences), CCR7 (150503, R&D Systems), CXCR3 (G025H7, Biolegend), CXCR5 (RF8B2, BD biosciences)), activation markers (Ki67 (B56, BD biosciences), CD38 (HIT2, BD biosciences)) and molecules associated with response to immunotherapy (PD-1 (EH12.2H7, Biolegend), TIGIT (MBSA43, eBioscience)). Cells were then washed in CyFACs buffer, before fixation and permabilisation with FIX & PERM Cell Fixation & Cell Permeabilization Kit (Thermo Fisher Scientific, U.S.A.) for 45 min on ice. Cells were, then incubated with a cocktail of antibodies reactive with intracellular antigens, including T-bet (4B10, BD biosciences), FoxP3 (PCH101, eBioscience) and CD247 (6B10.2, Biolegend), for 30 min on ice. Finally cells were fixed overnight in 4% PFA in PBS containing 1/4000 cell ID™ Intercalator-Ir (Fluidigm, U.S.A.), at 4°C.

Before data acquisition on the CyTOF 2 Helios upgraded mass cytometer, cells were washed in FACs buffer, and cell acquisition solution (CAS) (Fluidigm) prior to acquisition. Cells were re-suspended in CAS, at a concentration of 0.8 × 10^6^ cells/ml in a 1:10 solution of EQ beads (Fluidigm). Cells were acquired at a rate of 200–300 cells/s.

Data were normalised for intensity of EQ beads and analysed using FlowJo (version 10.6.1). Samples were pre-gated to remove beads and doublets, then de-barcoded based on their differently tagged CD45 antibodies and exported for further analysis. Manual gating was performed to identify PBMC subsets, based on the staining cocktails described above. Expression of additional surface proteins, including chemokine receptors, was determined by calculating the median signal intensity (MSI) within each of the gated subpopulations. The t-stochastic neighbourhood embedding (t-SNE) algorithm was applied using the FlowJo plugin. Files containing live non-neutrophil cells across a donor time series were down sampled without replacement using the FlowJo DownSample plugin and concatenated to ∼200000 events suitable for t-SNE analysis. The t-SNE algorithm was run using the 14 markers outlined above for major subset demarcation.

### Statistical analysis

Analysis was performed using GraphPad Prism 8 Software. Detailed descriptions included in the figure legends. One-way repeated measures ANOVA tests were performed to reveal changes due to different temperature and time-to-processing. For datasets with missing values, a one-way mixed effects ANOVA was performed. Linear regression after log transformation of neutrophil percentages was applied to the chemokine receptor data.

## Results

### Blood refrigeration prior to processing compromises viability and alters composition of recovered PBMC subsets

**Figure 1:**
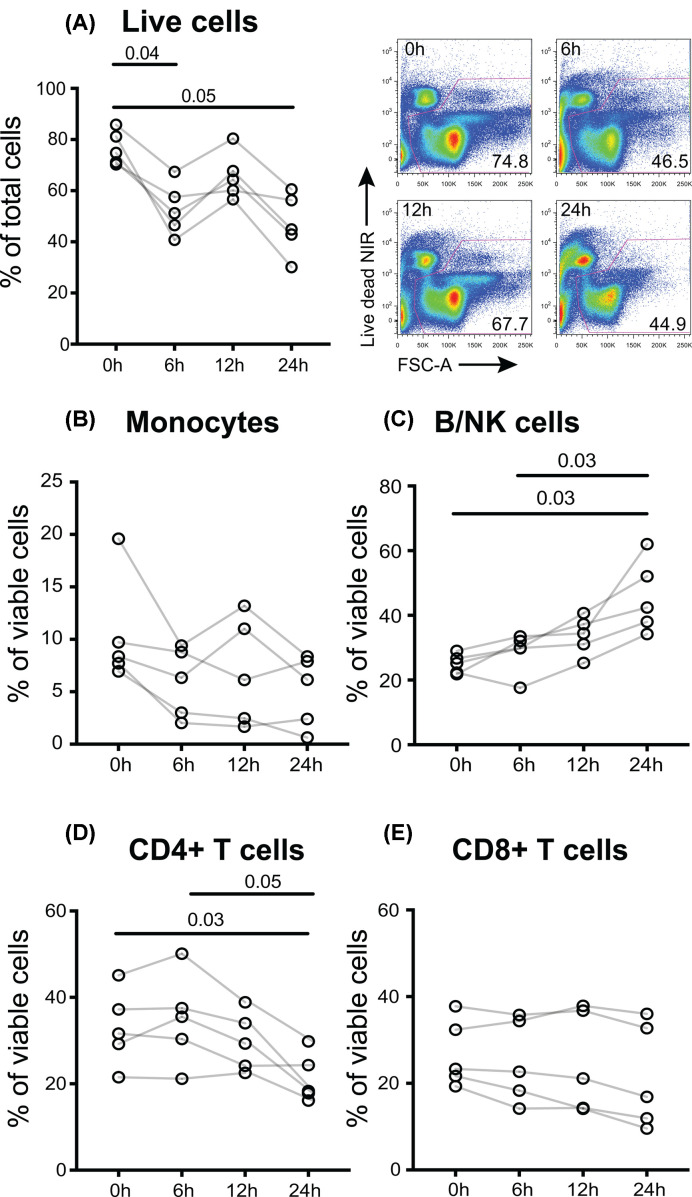
Effect of refrigerated storage of blood prior to PBMC processing A total of 4 × 5 ml EDTA blood tubes were collected from each of five donors. Tubes were processed immediately or refrigerated for 6, 12 or 24 h before processing. All PBMCs were cryopreserved and subsequently thawed and assessed by fluorescence flow cytometry for FSC and SSC to distinguish monocytes and lymphocytes, and expression of CD3, CD4 and CD14. (**A**) Viability was determined using NIR viability dye, with representative dot plots showing changes in forward scatter vs viability over time. Within viable cells, (**B**) monocytes were gated as CD4^lo^CD14^+^ and their identity confirmed by FSC/SSC, (**C**) B and NK cells were gated collectively as CD3-negative lymphocytes, (**D**) CD4^+^ T cells were gated as CD3^+^CD4^+^ and (**E**) CD8^+^ T cells were identified in this limited panel as CD3^+^CD4^−^. Data were analysed with a one-way repeated measures ANOVA with post-hoc Tukey’s tests and *P* values indicating significant differences between the timepoints are shown.

We initially set out to assess the effect of a delay in processing for refrigerated blood. Samples were processed after storage at 4°C for 6, 12 or 24 h, or freshly processed (within 2 h of blood draw). All PBMCs were cryopreserved at −80°C, then thawed for fluorescence flow cytometric analysis (Figure 1). The frequency of viable cells as determined by NIR viability dye staining decreased with storage at 4°C, with significant differences between the control and both the 6- and 24-h groups (Figure 1A). Individual cell populations showed differential effects of cold storage; CD3^+^CD4^+^ T cells were decreased, CD3^−^ lymphocytes (B and NK cells) were increased, and CD3^+^CD4^−^ T cells were unchanged. There was also a non-significant trend towards a reduction in the proportion of monocytes, determined as CD4^lo^CD14^+^ (Figure 1B–E). These data confirm that when stored under refrigerated conditions, a delay in time-to-processing can significantly impact the PBMC populations present in cryopreserved samples.

### Cell recovery is reduced when blood is refrigerated prior to processing

**Figure 2:**
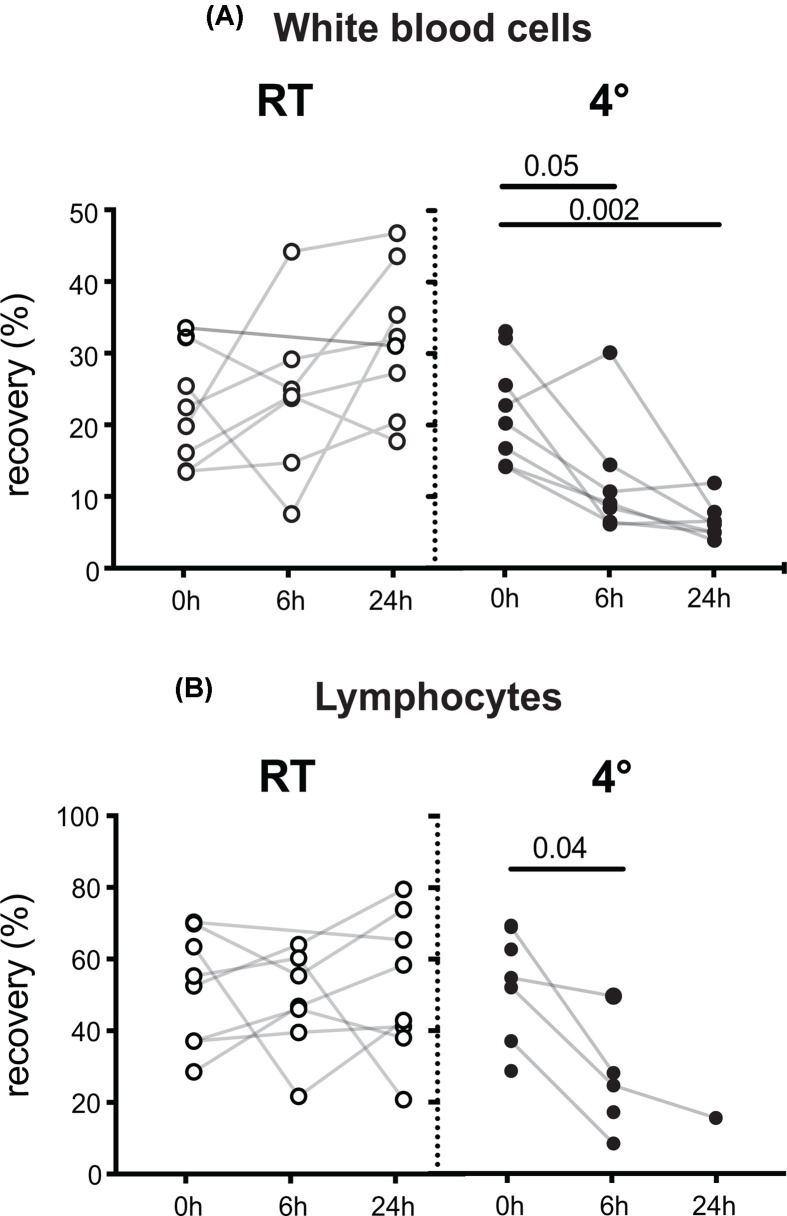
Effect of time and temperature prior to PBMC processing on recovery of total WBCs and lymphocytes A total of 5 × 7 ml heparin blood tubes were collected from each of seven donors, and 2 × 7 ml heparin blood tubes from an additional donor. Individual tubes were either processed immediately or kept at RT or 4°C for 6 or 24 h. PBMCs and an aliquot of whole blood were analysed using a Sysmex XP-300™ Automated Hematology Analyzer. These full blood counts were analysed using a one-way mixed effects ANOVA with post-hoc Tukey’s tests. A small drop in WBC was seen with RT storage (5% at 6 h, 11% at 24 h, *P*=0.04) and a larger drop at 4°C (12% at 6 h *P*=0.003, 25% at 24 h *P*=0.002). For the additional donor, only the 0 and 24 h RT analyses were performed. Cell recovery of (**A**) WBC and (**B**) lymphocytes was calculated as a percentage of the cell number loaded on to the Ficoll-hypaque gradient. Missing values for lymphocytes are due to the inability of the Sysmex to resolve a distinct lymphocyte peak. Data were analysed using a one-way mixed effects ANOVA with post-hoc Tukey’s tests and *P* values indicating significant differences between the timepoints are shown. For the 4°C analysis, a 24-h lymphocyte count was obtained for only a single sample, so the 0- and 6-h timepoints were analysed using a paired values *t* test.

We next sought to understand which stages of the blood processing protocol contributed to these changes in viability and subset recovery, and whether these changes were also evident in samples stored at RT prior to processing. Cell recovery was determined by comparing the number of WBCs recovered from Ficoll-hypaque gradient separation with the number loaded on to the gradient, as determined from the pre-gradient WBC count. At RT, only a small decrease in pre-Ficoll WBC counts was seen (12% over 24 h), a loss of 25% was evident with 24 h of cold storage. After processing, a much greater loss of WBCs was seen for refrigeration times of 6 h (62%) and 24 h (74%), while a small increase for samples stored at RT did not reach statistical significance (Figure 2A). In response to refrigeration, we observed the formation of many cell clumps containing erythrocytes, which were not retained at the density interface during PBMC isolation (not shown), and this may account for the poor WBC recovery. Lymphocyte recovery showed the same trends (Figure 2B). These observations demonstrate that refrigeration adversely reduced WBC recovery.

### RT storage of whole blood leads to neutrophil contamination of PBMCs but no changes in major cell subset distribution

**Figure 3:**
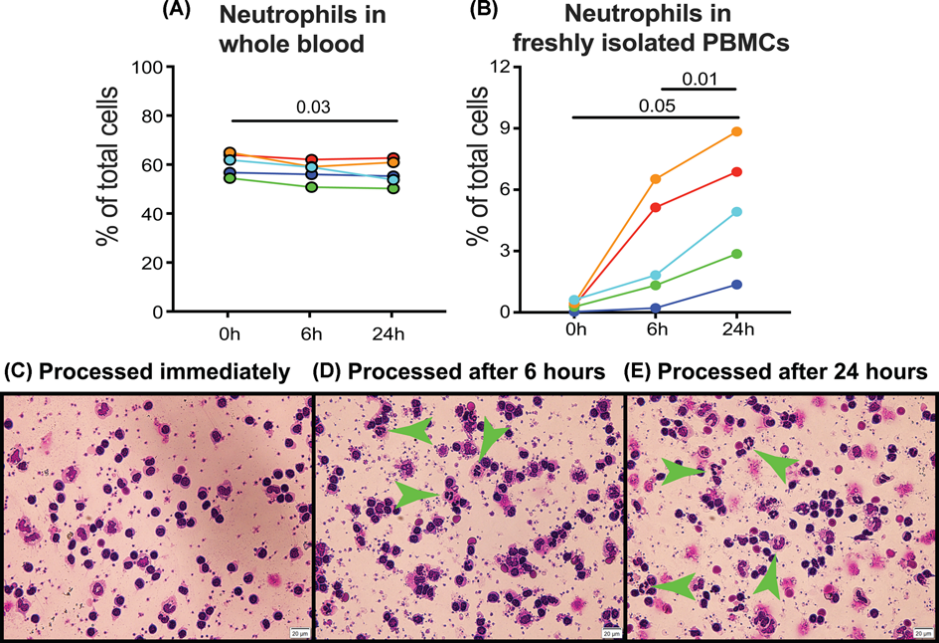
Effect of blood storage at RT on the number of neutrophils in PBMCs A total of 3 × 5 ml heparin blood tubes were collected from each of five donors, and individual tubes were either processed immediately or kept at RT for 6 or 24 h. Whole blood and PBMCs were analysed by fluorescence flow cytometry, using a cocktail of antibodies to CD3, CD4, CD8, CD56, CD14 and CD16. Dead cells were excluded with NIR viability dye. The percentage of neutrophils, identified by side scatter and expression of CD16, within live cells was calculated for (**A**) whole blood and (**B**) PBMCs. Each donor is indicated by a different colour. Data were analysed with a one-way repeated measures ANOVA with post-hoc Tukey’s tests and *P* values indicating significant differences between the timepoints are shown. (**C**–**E**) Aliquots of PBMCs were cytospun, stained with modified Giemsa and photographed. Green arrows indicate neutrophils with typical mature nuclear morphology in the 6 and 24 h samples.

Since WBC recovery was markedly reduced by refrigeration, we restricted further analysis to the effects of RT storage. We used fluorescent flow cytometry to assess changes in cellular composition of whole blood and PBMCs after 6 and 24 h RT. There was a slight decrease in the proportion of neutrophils in whole blood, while the proportion of neutrophils in PBMCs increased markedly after RT storage (Figure 3A,B). Flow cytometric identification of neutrophils by side scatter and expression of CD16 was confirmed histologically by identification of multilobular-nucleated neutrophils in cytocentrifuge images (Figure 3C–E).

**Figure 4:**
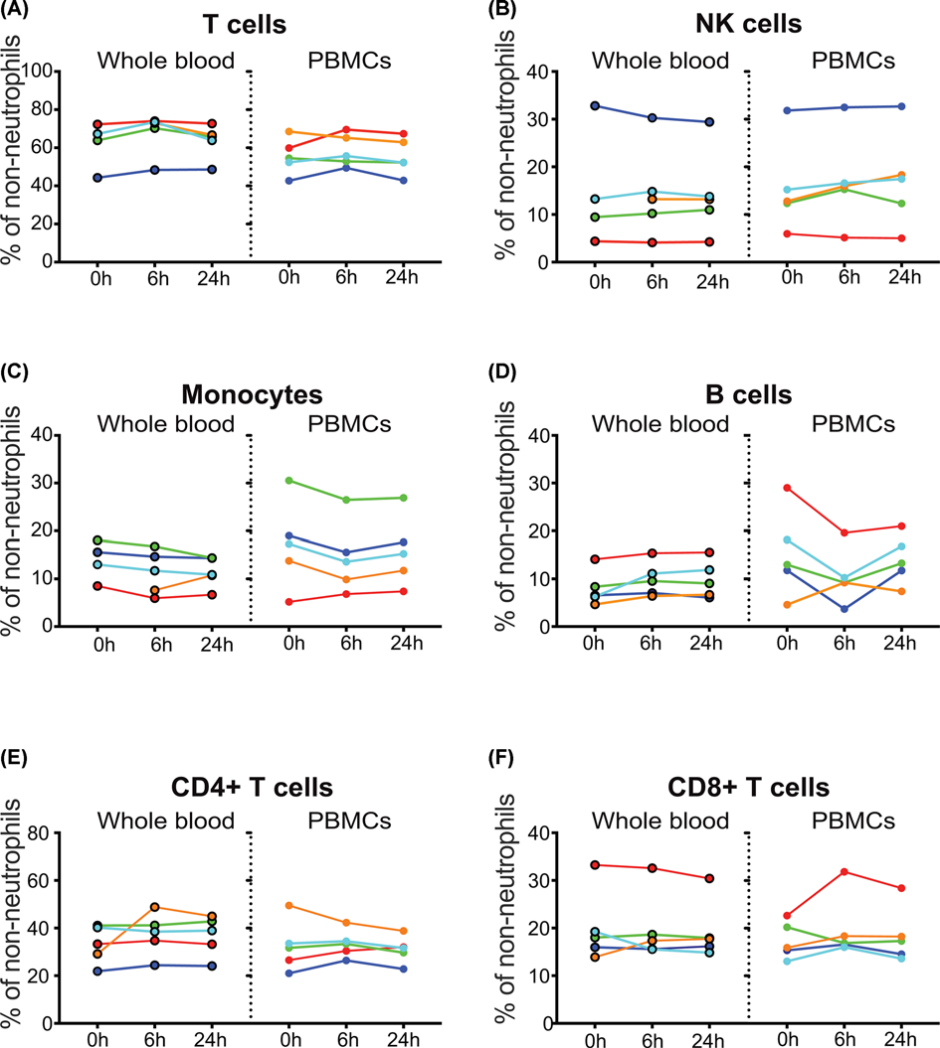
Effect of blood storage at RT on major cell subsets within PBMCs, assessed using fluorescence flow cytometry Data from the experiment described in Figure 3 were analysed to determine the percentage of major cell subsets, expressed as a proportion of non-neutrophil WBCs to account for the effect of low density neutrophil contamination in the 6 and 24 h samples. (**A**) Total CD3^+^ T cells, (**B**) CD56^+^ NK cells, (**C**) monocytes, gated for side scatter and CD14 expression, (**D**) CD19^+^ B cells, (**E**) CD4^+^ T cells and (**F**) CD8^+^ T cells. One-way repeated measures ANOVA revealed no significant differences between the timepoints for any of the cell subsets.

For the other major cell populations (monocytes, NK cells, B cells, T cells and their subsets CD4 and CD8 T cells), storage at RT had no significant effect on their representation within the non-neutrophil fraction of either whole blood or PBMCs (Figure 4). Similarly, viability assessment using Zombie NIR revealed no changes resulting from RT storage. In whole blood, all samples were over 99.4% viable, and in PBMCs the lowest viability recorded was 98.6% (data not shown). These data demonstrate that delaying the blood processing for an extended period of time leads to neutrophil contamination in PBMC preparations.

### Implementing mass cytometry for comprehensive immunophenotypic analysis

The results from fluorescence flow cytometric analysis of blood samples stored at RT suggested that a 24-h delay before processing would have minimal impact on the broad immunophenotype. However the type of immune signature that is increasingly being investigated in cancer patients includes a large number of smaller cell subsets that were not examined in the fluorescence analysis. Aliquots of the PBMC samples from the experiments shown in Figures 2-4 were cryopreserved and thawed for time of flight mass cytometric analysis.

**Figure 5:**
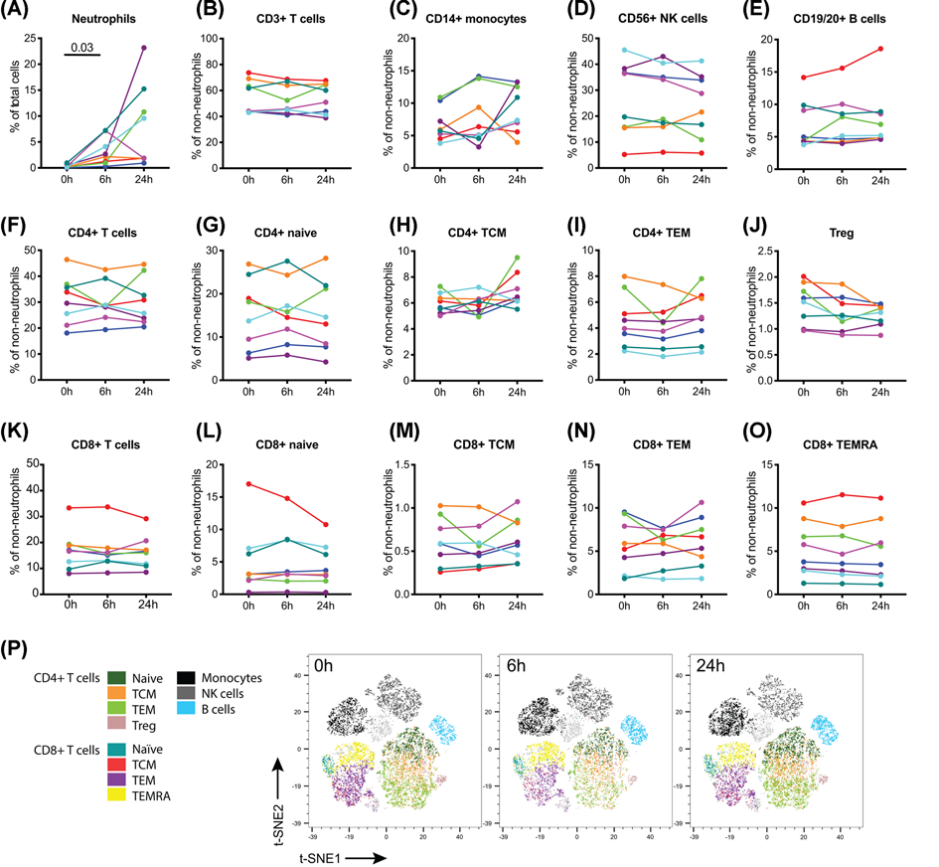
Effect of blood storage at RT on cell subsets within PBMCs, assessed using mass cytometry Cryopreserved PBMCs from the experiment described in Figure 3, plus an additional three donors, were thawed in two batches and analysed using mass cytometry. For each donor, the 0, 6 and 24 h samples were barcoded before staining in a single tube to ensure equivalence of staining conditions across timepoints. Data were gated for the populations indicated. Results for (**A**) neutrophils are expressed as proportion of total cells, while results for the other cell populations (**B–O**) are expressed as proportion of total non-neutrophil cells. Treg cells were gated as CD25^+^CD127^lo^ CD4^+^ T cells. Additional T cell subsets were gated for expression of CD45RA, CD45RO and CCR7 into naïve (CD45RA^+^CD45RO^−^CCR7^+^), TCM (CD45RA^−^CD45RO^+^CCR7^+^), TEM (CD45RA^−^CD45RO^+^CCR7^−^) and TEMRA (CD45RA^+^CD45RO^−^CCR7^−^) subsets. Each colour represents an individual donor. One-way repeated measures ANOVA revealed no significant differences between the timepoints for any of the cell subsets, apart from neutrophils. (**P**) Representative tSNE dimensionality reduction of non-neutrophils across time points for a donor with increasing neutrophil contamination over time (donor 3, represented in dark green in (A–O)). Dots represent individual cells and are coloured by populations in (B–O).

Comparison of the major cell populations identified by mass cytometric analysis of cryopreserved PBMCs (Figure 5) confirmed the results from the fluorescent cytometric analysis performed before cyopreservation (Figure 4). Monocytes, T, NK and B cell numbers were all stable, as were subsets of CD4 and CD8 T cells (Figure 5). CyTOF analysis of neutrophils (Figure 5A) confirmed the presence of low density neutrophils within PBMCs prepared after RT storage.

**Figure 6:**
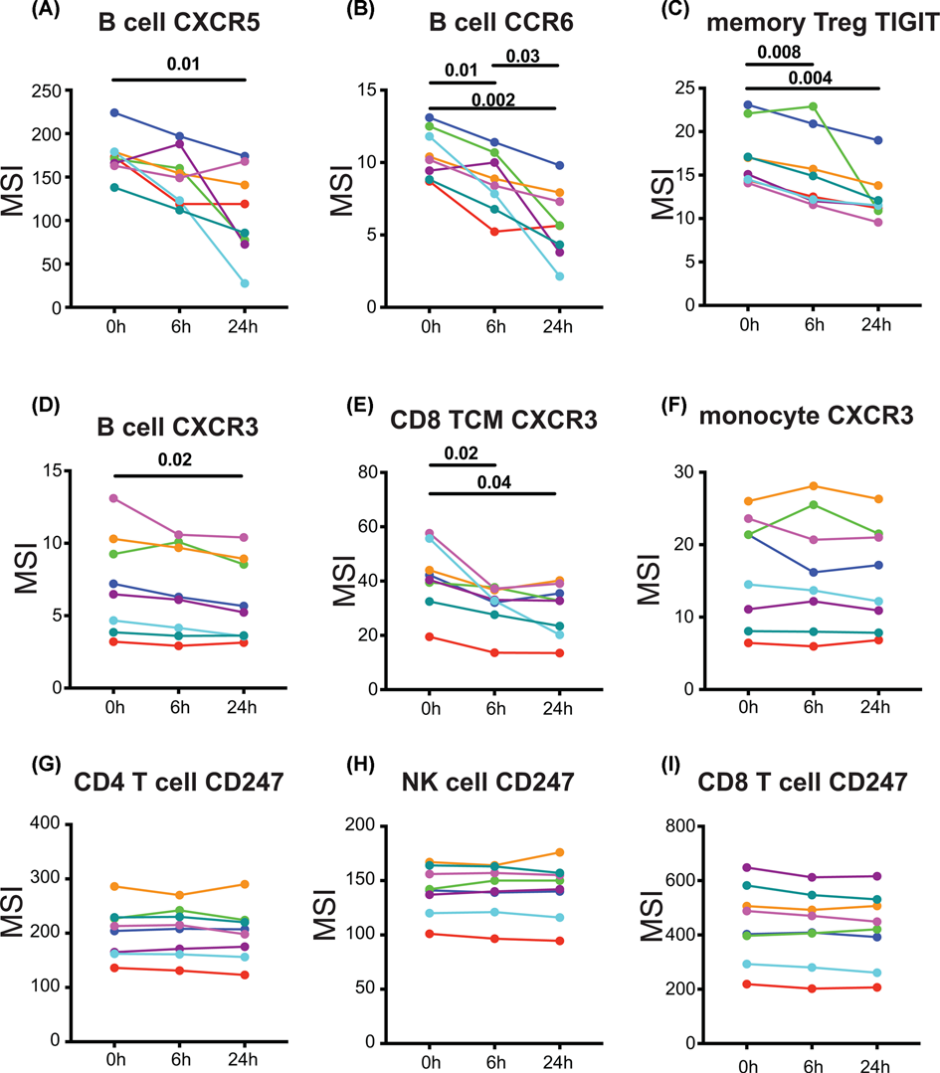
Effect of blood storage at RT on expression of cell surface proteins within PBMCs, assessed using mass cytometry Data from the experiment described in Figure 5 were analysed to determine the effect of RT blood storage on expression of cell surface proteins detected by metal-labelled antibodies. No proteins increased expression at 6 or 24 h. Proteins whose expression decreased significantly over time, as assessed by MSI, are illustrated in (**A**–**E**). Calculation of MSI for mass cytometry data is useful only when at least 50% of any cell population have detectable signals in that channel. For this reason, cell subsets with relatively high expression of the indicated proteins are shown. For (A–C) CXCR5, CCR6 and TIGIT, expression on all cell subsets was reduced, while for CXCR3, there was a disparity between (D) B cells, (E) CD8^+^ T cells and (**F**) monocytes. Data were analysed with a one-way repeated measures ANOVA with post-hoc Tukey’s tests and *P* values indicating significant differences between the timepoints are shown. (**G–I**) Expression of CD247 showed no significant change. Donors are identified by the same colours as in Figure 5.

More detailed analysis of the expression of individual cell surface proteins as detected by mass cytometry revealed time-dependent reductions in a subset of markers. These included the chemokine receptors CXCR5, CCR6 and CXCR3, and TIGIT. To illustrate these changes, MSIs for indicative cell subsets were calculated (Figure 6). Interestingly, expression of CD247 (TCRζ) was stable, in contrast with the findings in a previous publication [7].

### Reduction in chemokine receptor expression correlates with neutrophil number in PBMCs

**Figure 7:**
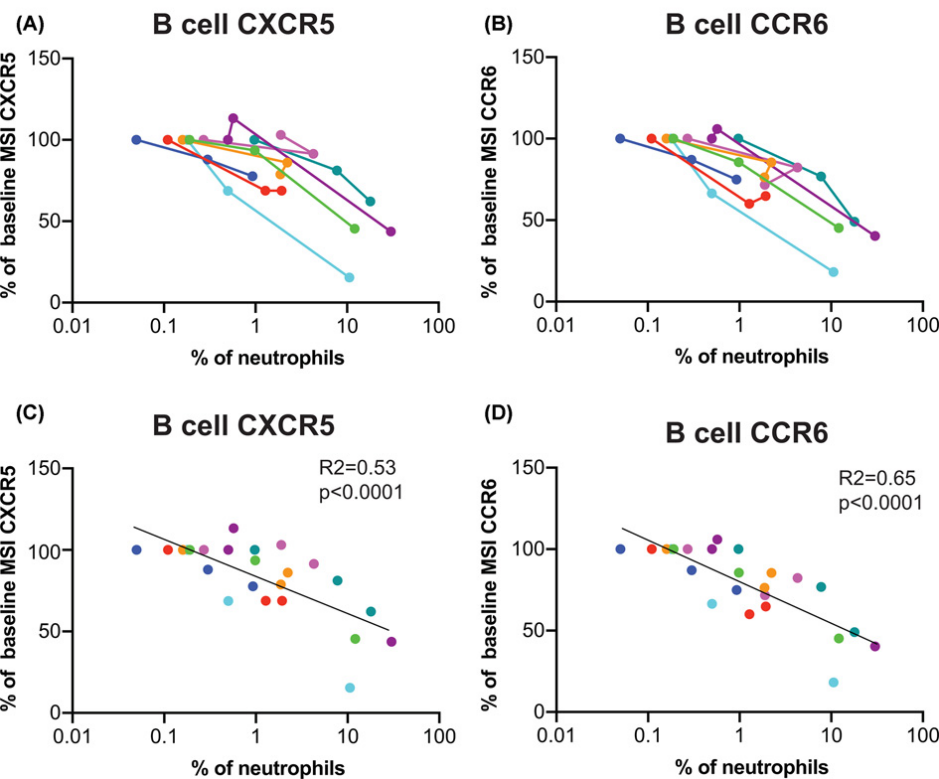
Correlation between number of low density neutrophils and reduction in expression of CXCR5 and CCR6 The data shown in Figure 6 were analysed as a function of the percentage of low density neutrophils within PBMCs. (**A**,**B**) MSIs for CXCR5 and CCR6 expression by B cells in PBMCs prepared after 6 or 24 h at RT storage were expressed as a percentage of values for 0 h samples and graphed against the percentage of low density neutrophils within PBMCs. Donors are identified by the same colours as in Figures 5 and 6. (**C**,**D**) Linear regression analysis of the correlation between increasing neutrophils and decreasing CXCR5 and CCR6.

Neutrophil contamination in PBMCs has previously been linked to changes in T-cell phenotype associated with whole blood storage before processing [7]. To test whether neutrophil contamination correlated with reduction in chemokine receptor expression, the change in expression of CXCR5 and CCR6 over time, expressed as a percentage of the value at time 0, was graphed relative to the percentage of neutrophils within each individual PBMC sample (Figure 7). There was a statistically significant association between the number of neutrophils contaminating the PBMCs and reduction in expression of CXCR5 and CCR6.

## Discussion

The present study provides a comprehensive analysis of the effects of whole blood sample storage conditions on multiparametric flow cytometry analysis of PBMC populations. With the increasing use of highly multiparametric flow cytometry, particularly mass cytometry, as a tool for analysis of clinical samples, factors that modify the data derived from cryopreserved PBMCs need to be documented and, if possible, avoided.

Storage of whole blood in the original collection tubes at 4°C for 6–24 h had major negative effects on viability of cryopreserved PBMCs (Figure 1). We observed a relative decrease in viability of CD4 T cells and an increase in CD3-negative lymphocytes, the majority of which are B and NK cells. An increase in NK cells and no change in B cells after 4°C storage before PBMC isolation has previously been documented in a study that also reported that NK cytotoxicity was well preserved at 4°C in acid citrate dextrose, but not heparinised tubes, illustrating how optimal storage conditions may differ for different cell populations within the same blood sample [10]. Profound reductions in recovery of both WBC and lymphocytes after cold storage (Figure 2) confirm that refrigeration of whole blood before preparation of PBMCs should be avoided when cells are required for cytometric analysis, since limiting the number of cells analysed can prevent the accurate quantification of rare but important populations. While the mechanism underlying cell loss in response to refrigeration is not well understood, we observed refrigeration-dependent formation of red cell-containing clumps that were not retained at the Ficoll-hypaque interface. It is possible that activation and associated aggregation of platelets due to low temperature may be responsible for the cell loss we observed at 4°C. The occurrence of spontaneous activation of platelets within whole blood at 4°C has been known for over 50 years [21,22], although it is also well established that 4°C storage of platelets is superior to either RT or 37°C in preserving their ability to respond to exposure to a range of activating stimuli [23,24]. Refrigeration before density gradient separation also led to an inability to resolve lymphocyte counts using the Sysmex XP-300™ Automated Hematology Analyzer, indicating that cold-exposure changed the resistive pulse sensing properties of PBMCs, which are used to measure the volume of WBC nuclei in Coulter counters such as the Sysmex. Although refrigeration prior to analysis is detrimental for subsequent determination of PBMC cell subset numbers, it may be preferable for assessment of soluble or secreted mediators, such as cytokines. This underscores the importance of study design in ensuring that sample collection is optimised for the planned analysis methods.

In contrast with refrigeration, RT storage for up to 24 h had no negative effects on cell recovery or viability. Indeed, there was a small increase in WBC recovery after 24-h storage at RT, attributable to co-purification of low density neutrophils with PBMCs, as reported by McKenna et al. [7]. We confirmed that neutrophil numbers in PBMCs increase with storage time at RT (Figure 3). Examination of the morphology of these low density neutrophils indicated that they had the nuclear conformation typical of mature neutrophils, and thus represent activated mature neutrophils rather than the immature neutrophils that have been described in inflammatory disease states [17,18,20]. Comparison of the proportions of neutrophils within RT stored PBMCs from donors who provided two blood samples at an interval of ∼6 months indicated that the degree of neutrophil contamination was highly variable, although for a single blood draw, it was usually higher after 24 h at RT, compared with 6 h.

To determine whether delayed processing at RT differentially affected the representation of non-neutrophil cell types within PBMCs, flow cytometry of whole blood and freshly isolated PBMCs from five donors was performed, and the results for each cell type were expressed as a proportion of non-neutrophil WBCs. No significant changes were seen in monocytes, NK cells, B cells, monocytes, CD4 T cells, CD8 T cells or total T cells (Figure 4). To test whether a more detailed analysis would reveal changes within these major cell types, cryopreserved aliquots of PBMCs from these five donors, plus those from an additional three donors, were analysed by mass cytometry (Figure 5). This confirmed the effect of RT storage on neutrophils, and extended the finding of stable numbers of major cell populations within non-neutrophil PBMCs to further subpopulations of T cells, including naïve, central memory and effector memory CD4 T cells, Tregs, and naive, central memory, effector memory and CD45RA-positive effector memory CD8 T cells.

However, a more detailed analysis of the level of expression of a number of cell surface molecules expressed by monocytes, NK cells, B cells and T cells indicated a time-dependent reduction with RT storage before processing. These included chemokine receptors and molecules implicated in patient responsiveness to cancer (Figure 6A–F). Interestingly, CD247, which has previously been reported as undergoing down-regulation in PBMCs prepared after whole blood storage at RT [7] was unaffected (Figure 6G–I). Examination of whether the extent of neutrophil contamination was related to down-regulation of the chemokine receptors CXCR5 and CCR6, which showed the largest effect of RT storage, indicated a highly significant correlation.

Neutrophil incubation with PBMCs has been shown to down-regulate expression of CD247 by T cells, leading to the conclusion that down-regulation of CD247 during RT storage of whole blood is directly attributable to neutrophil activation [7]. However, since we detected no CD247 down-regulation, the mechanism underlying the receptor down-regulation in our study may be different. Neutrophil activation and changes in receptor expression may both result from RT blood storage, without one causing the other. The correlation between the two phenomena does, however, suggest that they may both result from a third, upstream mechanism.

Several possible mechanisms have been reported previously. Firstly, platelet activation may be responsible for the observed changes in chemokine receptor expression. Upon activation, platelets are able to release chemokines that may inhibit or activate certain immune cells [25]. Activation of platelets is known to occur in whole blood at RT [26], although our study indicates that at RT it does not cause the cell clumping that we observed at 4°C. Activated platelets are able to activate neutrophils *in vivo* [27], although the extent to which this may occur in stored blood samples is unknown.

While not tested in the present study, it is important to consider ways to reduce effects of delayed processing. Studies have shown that diluting whole blood 1:1 in media such as PBS [7] or RPMI [8] prior to storing at RT could at least partially reverse the negative effects of blood storage. Dilution at the time of venesection reduced low density granulocyte contamination of PBMCs and preserved some aspects of T cell function. Gentle agitation has also been shown to reduce the negative impact of blood storage [8]. Interestingly, another study in which samples were shipped overnight demonstrated no negative effects on TCR signalling in PBMCs [12]. It may be difficult to fully control for the degree of agitation during shipping, but blood collection protocols incorporating immediate dilution could certainly be adopted at sites where PBMC preparation is not possible.

Overall, the present study was able to demonstrate effects of blood storage conditions on PBMC recovery after Ficoll-hypaque gradient separation, and to provide a detailed analysis of the effects on the results of subsequent mass cytometric analysis. We concluded that while the effects on PBMC composition of RT storage are far less profound than those of refrigeration, the contaminating low density neutrophils found in the PBMC layer need to be considered in analysis strategies. Furthermore, down-regulation of a variety of cell surface receptors after RT storage for between 6 and 24 h indicates that caution must be applied to measurements based on PBMCs that were not processed immediately after blood draw. For this reason, disclosure of temperature- and time-to-processing is important when publishing data from clinical trial PBMCs. Further work is needed to test whether these effects can be reversed by protocol changes such as dilution at the point of venesection.

## Data Availability

The data underlying this article will be shared on reasonable request to the corresponding authors.

## Abbreviations

CAS: cell acquisition solution
CyTOF: Cytometry by Time of Flight
FBS: foetal bovine serum
NIR: near-infrared
NK cell: natural killer cell
MSI: median signal intensity
PBMC: peripheral blood mononuclear cell
PBS: phosphate buffered saline
PFA: paraformaldehyde
RT: room temperature
Treg: Regulatory T cell
t-SNE: t-stochastic neighbourhood embedding
WBC: white blood cell
